# Supplementary material for: Future Proteins: Sustainable Diets for Tenebrio molitor Rearing Composed of Food By-Products
Source: Foods. 2023 Nov 11;12(22):4092. doi: 10.3390/foods12224092 (PMC10670904; doi:10.3390/foods12224092)
Supplement: Supplementary file 1 [file foods-12-04092-s001.zip › foods-2634697-supplementary.pdf]

Supplementary Materials

Comparison of x by group
(No adjustment)

Col Mean-<br>Row Mean	B_Box1	B_Box2	CGO_Box1	CGO_Box2	CN_Box1	CN_Box2
B_Box2	-0.814572<br>0.2077					
CGO_Box1	-2.404350<br>0.0081*	-1.655810<br>0.0489				
CGO_Box2	-1.812826<br>0.0349	-1.064286<br>0.1436	0.550271<br>0.2911			
CN_Box1	-3.200166<br>0.0007*	-2.572050<br>0.0051*	-1.122734<br>0.1308	-1.593958<br>0.0555		
CN_Box2	-3.521259<br>0.0002*	-2.893142<br>0.0019*	-1.427566<br>0.0767	-1.898790<br>0.0288	-0.270850<br>0.3933	
LSWBC	-1.170695<br>0.1209	-0.708049<br>0.2395	0.306375<br>0.3797	-0.048822<br>0.4805	1.074161<br>0.1414	1.288287<br>0.0988
MRP_Box1	-2.103568<br>0.0177*	-1.544776<br>0.0612	-0.296130<br>0.3836	-0.719730<br>0.2358	0.646836<br>0.2589	0.895386<br>0.1853
MRP_Box2	-1.196544<br>0.1157	-0.637751<br>0.2618	0.573964<br>0.2830	0.150365<br>0.4402	1.436044<br>0.0755	1.684594<br>0.0460
SO_Box1	-1.014145<br>0.1553	-0.207598<br>0.4178	1.450941<br>0.0734	0.864350<br>0.1937	2.396173<br>0.0083*	2.715373<br>0.0033*
SO_Box2	-1.102047<br>0.1352	-0.295500<br>0.3838	1.370041<br>0.0853	0.783450<br>0.2167	2.328121<br>0.0100*	2.647321<br>0.0041*
SP	-1.866874<br>0.0310	-1.096848<br>0.1364	0.561994<br>0.2871	-0.001866<br>0.4993	1.620953<br>0.0525	1.931251<br>0.0267
WB_Box1	-1.500235<br>0.0668	-0.941443<br>0.1732	0.282637<br>0.3887	-0.140961<br>0.4440	1.171801<br>0.1206	1.420350<br>0.0778
WB_Box2	-0.572964<br>0.2833	-0.014172<br>0.4943	1.172155<br>0.1206	0.748556<br>0.2271	1.978625<br>0.0239*	2.227174<br>0.0130*
WBc	-1.915764<br>0.0277	-1.416942<br>0.0782	-0.303225<br>0.3809	-0.684464<br>0.2468	0.558342<br>0.2883	0.785902<br>0.2160
WK_Box1	-1.004334<br>0.1576	-0.255794<br>0.3991	1.302379<br>0.0964	0.752108<br>0.2260	2.238025<br>0.0126*	2.542857<br>0.0055*
WK_Box2	-0.740546<br>0.2295	0.007993<br>0.4968	1.547771<br>0.0608	0.997499<br>0.1593	2.448166<br>0.0072*	2.752998<br>0.0030*

Col Mean-<br>Row Mean	LSWBC	MRP_Box1	MRP_Box2	SO_Box1	SO_Box2	SP
MRP_Box1	-0.489048<br>0.3124					
MRP_Box2	0.154209<br>0.4387	0.733427<br>0.2316				
SO_Box1	0.587077<br>0.2786	1.394402<br>0.0816	0.491615<br>0.3115			
SO_Box2	0.536817<br>0.2957	1.333786<br>0.0911	0.430999<br>0.3332	-0.087052<br>0.4653		
SP	0.048127<br>0.4808	0.728683<br>0.2331	-0.153955<br>0.4388	-0.890726<br>0.1865	-0.807544<br>0.2097	
WB_Box1	-0.061166<br>0.4756	0.487860<br>0.3128	-0.245567<br>0.4030	-0.793887<br>0.2136	-0.733272<br>0.2317	-0.141571<br>0.4437
WB_Box2	0.596449<br>0.2754	1.237658<br>0.1079	0.504231<br>0.3070	0.129050<br>0.4487	0.189665<br>0.4248	0.760768<br>0.2234
WBc	-0.488811<br>0.3125	-0.031829<br>0.4873	-0.710850<br>0.2386	-1.283745<br>0.0996	-1.229583<br>0.1094	-0.691147<br>0.2447
WK_Box1	0.534306<br>0.2966	1.298703<br>0.0970	0.428607<br>0.3341	-0.062599<br>0.4750	0.018299<br>0.4927	0.772547<br>0.2199
WK_Box2	0.692705<br>0.2442	1.487605<br>0.0684	0.617510<br>0.2684	0.198988<br>0.4211	0.279887<br>0.3898	1.023999<br>0.1529

Col Mean-<br>Row Mean	WB_Box1	WB_Box2	WBc	WK_Box1
WB_Box2	0.749798<br>0.2267			
WBc	-0.483499<br>0.3144	-1.177678<br>0.1195		
WK_Box1	0.719934<br>0.2358	-0.169582<br>0.4327	1.205540<br>0.1140	
WK_Box2	0.908836<br>0.1817	0.019319<br>0.4923	1.375552<br>0.0845	0.245391<br>0.4031

Figure S1. Results (*p*-values) of Dunn´s test, comparing among different feeding trials.

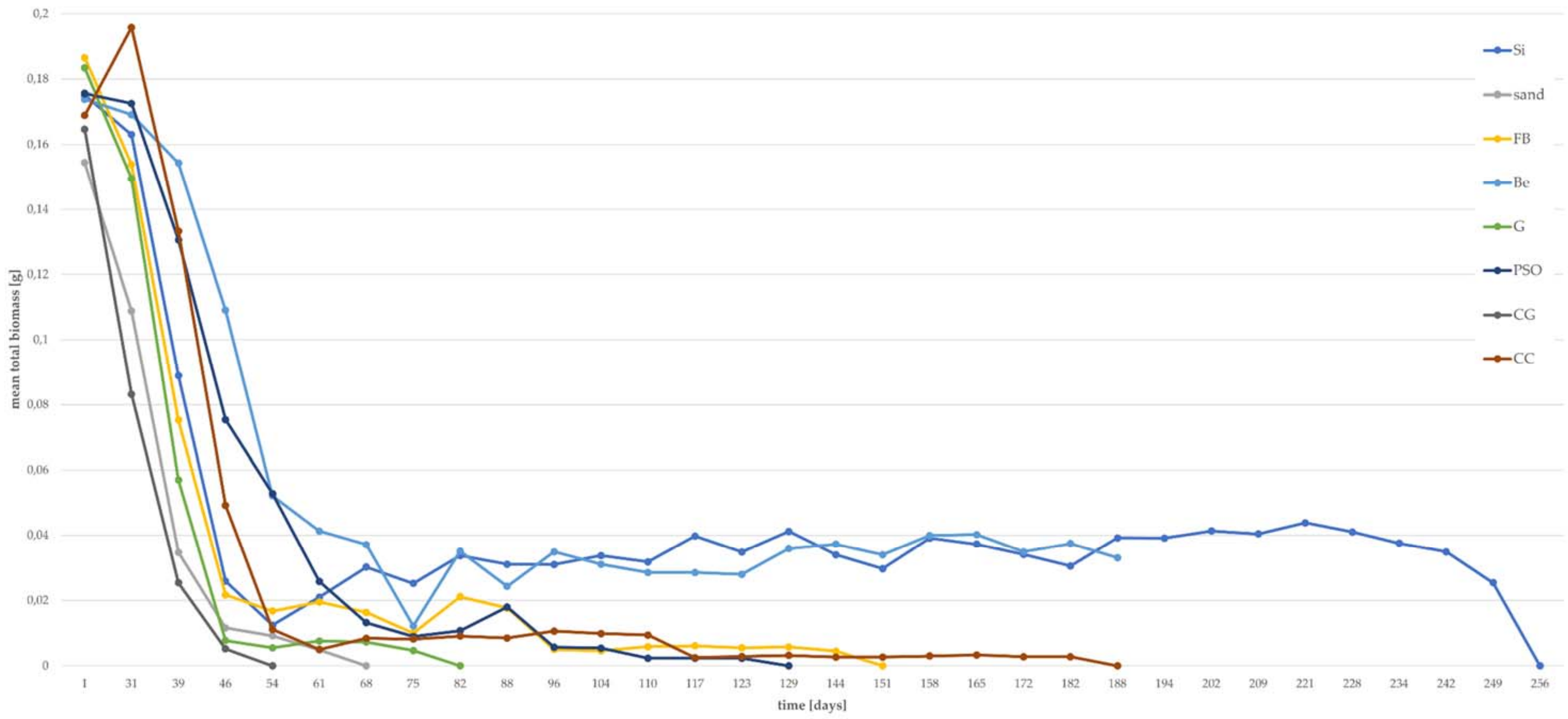

Figure S2. Mean total biomass of “not recommended diets” in gram, determined weekly. (n = 100 for Si, FB, Be, G, PSO, CG, CC; n = 50 for sand).

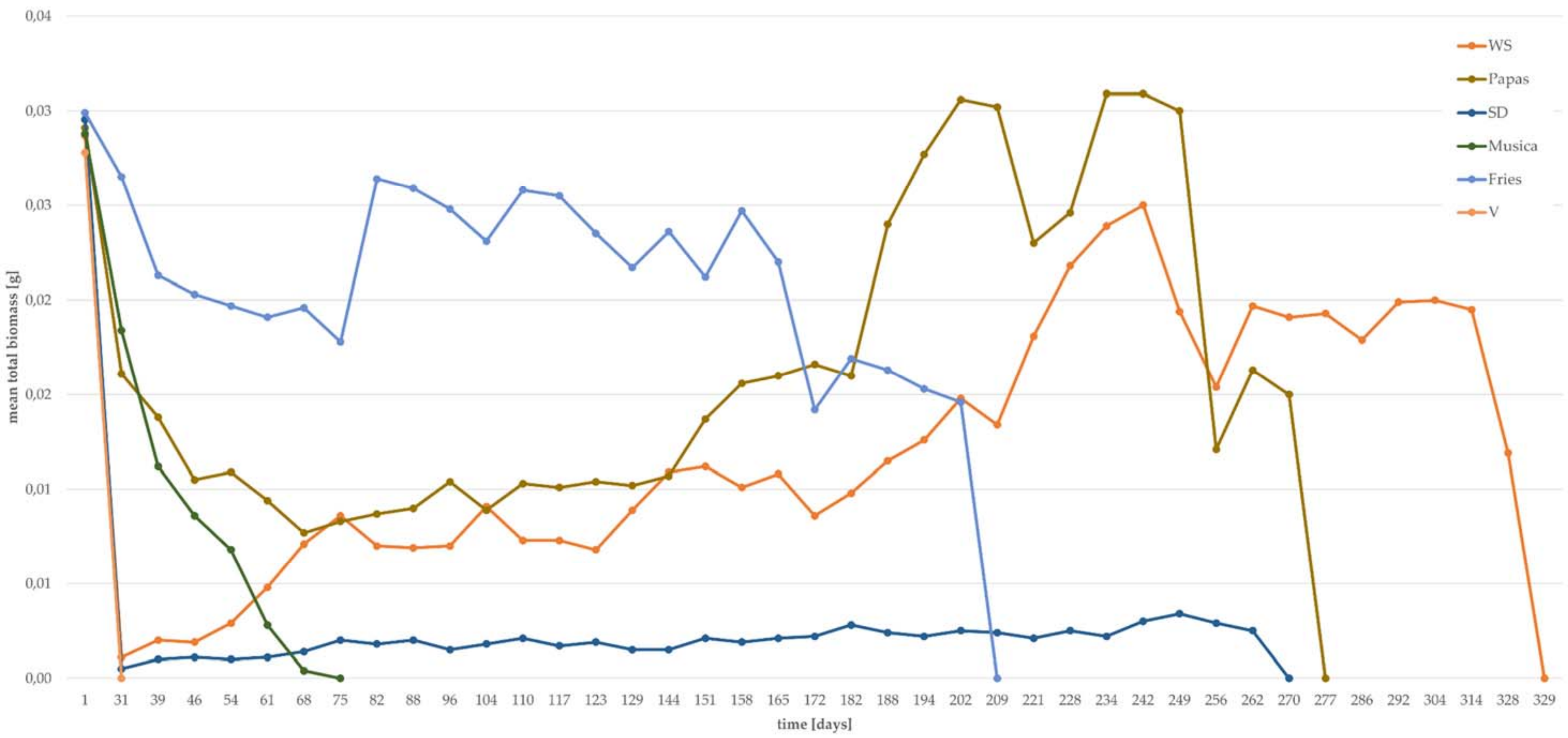

Figure S3. Mean total biomass of “not recommended diets” in gram, determined weekly. (n = 100 for WS, SD; n = 50 for Papas, Fries, V, Musica)

Table S1. Applied single substrate diets for *T. molitor* feeding trials.

	Feed	Abbreviation	Substrate specification	Source	Species	Further processing
1	wheat bran*	WB		Farina-Mühle, Raaba; Uitz-Mühle, Knittelfeld	*Triticum* sp.	-
2	malt residual pellets*	MRP		STAMAG Stadlauer Malzfabrik GesmbH; Graz		cut
3	corn germ meal, organic*	CGo		Lerchenmühle, Wieser GmbH; Golling	*Zea mays*	-
4	coffee grounds †	CG		coffee kitchen; FH Joanneum Graz	*Coffea* sp.	dried
5	coffee chaff †	CC		Albin Sorger "zum Weinrebenbäcker" GmbH, Graz	*Coffea* sp.	cut
6	pumpkin kernel cake †	PSO		Haindl Mühle, Kalsdorf bei Graz	*Cucurbita pepo* subsp. *pepo* var. styriaca	-
7	pearl oyster mushroom mycelia with coffee grounds and coffee chaff	AK		Pilzkiste GESBR, Graz	*Pleurotus ostreatus*	dried
8	*Fallopia* x *bohemica* †	FB	dried rhizome	René Rehorska	Hybride of *F. japonica* and *F. sachalinensis*	dried
9	Sida †	Si	dried whole plant	Simon Berner	*Sida hermaphrodita*	-
10	sweet chestnuts with peel*	CN	“Bruch”	Stefan Pichler	*Castanea sativa*	-
11	brewer´s spent grain	D		Brauerei Puntigam, Graz		frozen, dried
12	garlic peel †	G		coffee kitchen; FH Joanneum Graz	*Allium sativum*	cut
13	bread remains*	B	remains of not sold baked goods (not sweet)	Bakeries, Graz		dried, cut
14	runner beans †	Be	“Bruchbohnen”	René Rehorska	*Phaseolus coccineus*	cut

15	Mur sand †	sand	sand from the riverbank of the Mur in Mureck	René Rehorska		-
16	hempseed cake	WC	residue from pressing the oil	Simon Berner	*Cannabis sativa*	cut
17	acron flour	EH	flour made out of oak fruit nuts	Nadja Aldrian	*Quercus* spp.	-
18	sawdust †	SD		Farm near Graz	*Picea abies*	-
19	Soybeans*	SO		Iglo Austria	*Glycine max*	dried, cut
20	Urtica*	U		Forest near Graz	*Urtica dioica*	dried
21	foam peanuts	V	packing material			cut
22	pearl oyster mushroom	A	remaining fungus stipes after harvesting	Pilzkiste GESBR, Graz	*Pleurotus ostreatus*	dried
23	wheat straw †	WS	combine harvester remains	Farm near Graz	*Triticum* sp.	cut
24	sweet potatoes*	SP		Local retailer: Merkur	*Ipomoea batatas* "Beauregard"	dried, cut
25	potatoes	Maestro		Local retailer: Spar	*Solanum tuberosum* "Maestro"	dried, cut
26	potatoes †	Musica		Local retailer: Spar	*Solanum tuberosum* "Musica"	dried, cut
27	potatoes †	Fries		René Rehorska	*Solanum tuberosum* "Friesländer"	dried, cut
28	jerusalem artichoke †	Papas		Local retailer: Merkur	*Helianthus tuberosus* "Papas"	dried, cut
29	wheat germs (extracts)*	WK		Longevity Labs, Graz; Nico Teuschler		dried

*Successful substrate, † deadly substrates

Table S2. Nutrient composition of different substrates.

	WB	MRP	PSO	D	CG	CC	CGo[1]	AK	Si	B	CN	WC	Be	SP	Papas	WK	U	WS	TM[2]	V
**Dry matter**	922	901	948	958	916	954	900	914	912	889	918	924	882	881	921	921	923	921	957	886
**nutrients** (g/kg; DM)																				
crude protein	194	191	645	294	148	176	148	176	18	163	80	353	257	90	153	382	209	55	597	9
Crude fat	59	30	147	133	166	37	111	21	5	36	31	79	25	10	8	103	25	12	273	nn
crude fiber	117	110	34	178	259	350	70	151	605	8	40	323	50	53	43	-	8	-	-	-
Crude ash	62	52	90	47	16	72	30	178	33	29	25	75	46	65	68	-	-	-	-	-
Starch	178	294	27	39	32	67	429	25	29	604	394	11	404	391	nn	236	-	nn	nn	533
Sugar	64	80	38	13	5	8	42	10	8	63	194	28	80	258	647	115	59	68	15	10
Calcium	1.5	1.9	1.2	5.1	2.3	11.1	0.4	57.8	9.4	0.8	1.0	2.6	1.1	5.4	1.3	0.8	68.4	2.7	0.8	0.5
Phosphor	13.9	4.9	20.3	8.0	1.5	0.9	6.8	1.4	0.5	2.4	1.7	16.2	5.7	2.7	4.7	10.5	4.7	2.1	10.9	0.2
Magnesium	5.7	1.6	8.8	2.6	1.7	3.8	2.5	2.2	0.7	0.7	1.1	6.9	2.3	1.1	1.0	1.8	7.4	1.0	5.1	0.7
Kalium	14.1	10.7	15.8	0.4	3.9	18.6	10.2	8.8	4.4	3.4	10.9	11.9	19.3	27.6	30.2	7.6	28.2	18.5	10.4	0.6
Natrium	0.14	0.28	5.71	0.21	0.14	0.22	0.09	0.75	0.29	8.80	0.23	0.23	0.25	0.22	0.20	0.28	0.21	0.24	1.25	0.88
**micronutrients** (mg/kg; DM)																				
Iron	151.8	189.8	167.7	200.4	49.1	307.1	-	347.9	51.5	23.6	22.9	241.3	69.2	132.8	67.7	110.7	159.3	99.9	63.7	20.3
Manganese	144.3	35.5	72.8	60.5	31.7	51.4	-	85.3	29.6	16.9	148.1	153.7	20.4	10.2	6.3	175.9	78.0	21.7	16.7	0.8
Zinc	108.5	52.5	140.3	124.2	14.2	19.9	-	28.4	7.7	16.9	19.6	108.2	35.1	11.2	11.5	142.2	19.5	18.5	141.1	3.4
Copper	14.1	7.8	23.2	18.8	20.7	69.2	-	31.7	3.3	3.4	9.8	27.1	7.9	4.1	4.2	8.7	7.6	3.3	24.0	0.6

DM = dry matter; nn = successfully analyzed, value below detection limit; [1] analyzed by BOKU; [2] *Tenebrio molitor* larvae, fed with wheat bran. Blue color: successful substrates.
